# Modelling of magnetic microbubbles to evaluate contrast enhanced magnetomotive ultrasound in lymph nodes – a pre-clinical study

**DOI:** 10.1259/bjr.20211128

**Published:** 2022-05-19

**Authors:** Sandra Sjöstrand, Marion Bacou, Katarzyna Kaczmarek, Maria Evertsson, Ingrid K Svensson, Adrian JW Thomson, Susan M Farrington, Susan J Moug, Tomas Jansson, Carmel M. Moran, Helen Mulvana

**Affiliations:** Department of Biomedical Engineering, Faculty of Engineering, Lund University, Lund, Sweden; Colorectal Cancer Genetics Group, Cancer Research UK Edinburgh Centre, Institute of Genetics and Cancer, University of Edinburgh, Edinburgh, United Kingdom; Department of Biomedical Engineering, Faculty of Engineering, University of Strathclyde, Glasgow, United Kingdom; Department of Clinical Sciences Lund, Lund University, Lund, Sweden; Department of Biomedical Engineering, Faculty of Engineering, Lund University, Lund, Sweden; Edinburgh Preclinical Imaging, Centre for Cardiovascular Science, University of Edinburgh, Edinburgh, United Kingdom; Colorectal Cancer Genetics Group, Cancer Research UK Edinburgh Centre, Institute of Genetics and Cancer, University of Edinburgh, Edinburgh, United Kingdom; Consultant General and Colorectal Surgeon, Royal Alexandra Hospital, Paisley and Golden Jubilee National Hospital, Honorary Professor, University of Glasgow, Glasgow, United Kingdom; Department of Clinical Sciences Lund, Lund University, Lund, Sweden and Clinical Engineering Skåne, Digitalisering IT/MT, Skåne Regional Council, Lund, Sweden; University of Edinburgh, Edinburgh, United Kingdom; Department of Biomedical Engineering, Faculty of Engineering, University of Strathclyde, Glasgow, United Kingdom

## Abstract

**Objectives:**

Despite advances in MRI the detection and characterisation of lymph nodes in rectal cancer remains complex, especially when assessing the response to neoadjuvant treatment. An alternative approach is functional imaging, previously shown to aid characterisation of cancer tissues. We report proof of concept of the novel technique Contrast-Enhanced Magneto-Motive Ultrasound (CE-MMUS) to recover information relating to local perfusion and lymphatic drainage, and interrogate tissue mechanical properties through magnetically induced deformations.

**Methods:**

The feasibility of the proposed application was explored using a combination of experimental animal and phantom ultrasound imaging, along with finite element analysis. First, contrast-enhanced ultrasound imaging on one wild type mouse recorded lymphatic drainage of magnetic microbubbles after bolus injection. Second, tissue phantoms were imaged using MMUS to illustrate the force- and elasticity dependence of the magnetomotion. Third, the magnetomechanical interactions of a magnetic microbubble with an elastic solid were simulated using finite element software.

**Results:**

Accumulation of magnetic microbubbles in the inguinal lymph node was verified using contrast enhanced ultrasound, with peak enhancement occurring 3.7 s post-injection. The magnetic microbubble gave rise to displacements depending on force, elasticity, and bubble radius, indicating an inverse relation between displacement and the latter two.

**Conclusion:**

Combining magnetic microbubbles with MMUS could harness the advantages of both techniques, to provide perfusion information, robust lymph node delineation and characterisation based on mechanical properties.

**Advances in knowledge:**

(a) Lymphatic drainage of magnetic microbubbles visualised using contrast-enhanced ultrasound imaging and (b) magnetomechanical interactions between such bubbles and surrounding tissue could both contribute to (c) robust detection and characterisation of lymph nodes.

## Introduction

Colorectal cancer (CRC) can progress by lymphatic spread.^1^ Lymph node status has been identified as an important factor to consider during staging and subsequent treatment planning for CRC,^2^ and other metastatic tumours that typically spread in this manner.^3–6^ Reliable lymph node assessment is a deciding factor in confidently proposing neoadjuvant therapy or straight to surgery. Accurate lymph node staging could shift surgery from a major procedure to smaller resections,^7^ thereby reducing risk of complications, and potentially improving a patient’s quality of life. In CRC, all patients in the UK undergo contrast CT and MRI imaging during pre-treatment staging,^8^ but these techniques can be limited for the detection and characterisation of lymph tissue or extranodal tumour deposits, especially in relation to neoadjuvant therapy.^9^

Previous research indicates numerous potential markers that could indicate extranodal tumour deposits^10^ and lymphatic metastasis in CRC,^11^ and yet these cannot be easily interrogated using currently available diagnostic measures. In particular, these include swelling^12^ of metastasised lymph nodes and alterations to tissue stiffness, where changes may relate to cancer progression and metastasis.^13^ For example, endoscopic ultrasound can be used to assess the primary colorectal tumour and lymph node status in suspected early stage cancers^8^ with infiltrated lymph nodes appearing as round, hypoechoic regions that are larger than 5 mm in size.^14,15^ However, this method of differentiating metastatic lymph nodes is highly operator-dependent,^16^ and indeed no single parameter has sufficient diagnostic performance.^17^ This points to a need for a more reliable and robust method for pre-treatment staging and risk stratification of CRC, specifically for locating and evaluating lymph nodes and tumour deposits.^9^

Microbubbles have been used as an ultrasound contrast agent for decades,^18^ and have recently been applied for sentinel lymph node localisation.^19–23^ Microbubbles have several interesting properties related to biomedical application; in addition to providing contrast, they have the potential to be used for targeted delivery^24^ and reversible opening of cellular membranes by sonoporation.^25^

Magnetomotive ultrasound is a pre-clinical technique where the contrast mechanism is linked to the mechanical properties of the interrogated tissue and can be used to locate sentinel lymph nodes.^26^ A contrast agent consisting of nanometre-sized magnetic particles, injected subcutaneously, accumulates in the draining lymph node, where it can be excited using a magnetic field which gives rise to oscillations and consequent tissue displacement. The tissue movement can be detected using ultrasound when a concurrently registered ultrasound sequence is used and if the interaction between the contrast agent and the external magnetic field is sufficiently strong. One factor that influences the volumetric magnetic force and thereby the displacement amplitude is the configuration of the magnetic material.^27^ The response also depends on the viscoelastic properties of the medium in which the contrast agent is located.^28,29^ This makes it possible to extract information about tissue mechanical properties from the magnetomotive signal.^30^ These properties suggest that magnetomotive ultrasound has potential to be used for tissue stiffness sensing of lymph nodes.

In order to harness the useful properties of both microbubbles and MMUS, we propose using magnetic microbubbles for contrast-enhanced magnetomotive ultrasound (CE-MMUS). Specifically, the configuration of magnetic nanoparticles in a microbubble shell would generate a different mechanical response to a magnetic force and could enhance sensitivity, as compared to MMUS using magnetic nanoparticles. In the original technique, the magnetic force is transferred directly from magnetic nanoparticles to the surrounding soft tissue. In the suggested novel technique, the magnetic nanoparticles are incorporated in a microbubble shell, and the force transferred via this shell. We hypothesise that magnetic microbubbles could be used to interrogate tissue mechanical properties through magnetically induced deformation but can also be used to recover additional information relating to local perfusion and lymphatic drainage.

In this proof of concept study, we explore the feasibility of these scenarios using a combination of experimental ultrasound imaging and finite element analysis (FEA), a versatile numerical tool that has been applied extensively to the modelling of microbubbles in ultrasound fields.^31,32^ Each method is used to explore different integral aspects of the proposed technique, including delivery of the contrast agent to the tissue of interest, and mechanical interaction between the microbubble and tissue. First, contrast-enhanced lymph node imaging is experimentally tested using magnetic microbubbles. The second experimental section then examines the dependence of MMUS displacement amplitude on instantaneous Young’s modulus and applied force magnitude in a tissue phantom. These dependencies could be exploited to gain clinically relevant insights into tissue mechanical properties from MMUS and CE-MMUS data. Experimental measurements are also used to estimate the magnetic force magnitude, which is incorporated in a finite element model. This model is used to investigate the magnetomechanical and contact interactions of one magnetic microbubble and an elastic solid, representing the interior of a lymph node capsule. The simulation illustrates how a magnetic force can be used to: (a) manipulate a magnetic microbubble and (b) induce a displacement in an elastic solid, and thereby (c) assess the feasibility of CE-MMUS and elastography in the lymphatic context. Simulations of the CE-MMUS response are compared and contrasted with an analytical solution^33^ that captures the response in relation to Young’s modulus, applied force and microbubble radius. These simulations together with the experimental work will indicate if these fundamental requirements for CE-MMUS are met, and if so, also indicate interesting research avenues.

## Methods

Two separate experiments were carried out to demonstrate contrast-enhanced and magnetomotive ultrasound. The combination of the two, CE-MMUS, was then investigated by exploring the magnetomechanical interactions between a magnetic microbubble and an elastic solid using FEA.

In the following sections, we present the experimental methodologies, including the preparation and imaging of magnetic microbubbles, preparation and MMUS imaging of a tissue phantom material, and mapping of the magnetisation field. We then introduce the finite element model, starting with geometry, contact and load definitions, structural mechanics and finally report solver configuration and convergence analysis.

## Experimental methods

Contrast-enhanced ultrasound imaging was preformed to verify that magnetic microbubbles could accumulate and be imaged in the lymph node.

### Magnetic microbubble preparation

Magnetic nanoparticles were attached to the surface of microbubbles through biotin−streptavidin interaction as previously described.^24^ Magnetic nanoparticles (FluidMAG-CMX, 50 nm, chemicell, Berlin, Germany), were biotinylated and conjugated with MicroMarker (FUJIFILM VisualSonics Inc. Toronto, Canada) that had been prepared per instruction for bolus injection to a concentration of 2*10^15^ microbubbles m^−3^ (2*10^9^ microbubbles per ml). 4.2*10^−8^ m^3^ (4.2 µl) of 12.5 kg m^−3^ (mg/ml) of biotinylated magnetic nanoparticles were added to one vial of MicroMarker, yielding approximately 35 µg iron per 5*10^−8^ m^3^ (50 µl) injection. Successful conjugation using this procedure could be confirmed by observing the suspension in the presence of a permanent magnet, and yields microbubbles that can be magnetically retained under flow.^24^ The magnetic microbubble suspension was prepared immediately prior to use.

### Contrast-enhanced ultrasound imaging in animal model

All animal experiments were approved by the University of Edinburgh Animal Welfare and Ethical Review Body and were conducted in accordance with the Animal (Scientific Procedures) Act UK 1968, and performed under a UK Home Office project license (P02F16F82). To determine suitability of the magnetic microbubbles for ultrasound contrast imaging of lymph nodes, a study was performed in a wild type mouse. Procedures were carried out under isofluorane anaesthesia using 100% O_2_ at a rate of 1.7*10^−8^ m^3^s^−1^ (1 ml min^−1^) with an initial induction at 4% isofluorane, then maintained at 2% thereafter. Heart rate and body temperature were monitored and remained within normal ranges throughout. A 5*10^−8^ m^3^ (50 µl) bolus of magnetic microbubbles were injected in the tail vein while imaging (Vevo 3100, FUJIFILM VisualSonics, Toronto, Canada) the inguinal lymph node at a frame rate of 25 Hz using the MX250 transducer with centre frequency 20 MHz operating in non-linear contrast mode. No external magnetic field was applied, and the acquisition time was 30 s. The animal was sacrificed whilst anaesthetised directly post-imaging.

### Tissue mimicking material

In addition to contrast-enhanced ultrasound imaging of the mouse lymph node, supporting experimental MMUS data were produced from tissue mimicking phantoms to inform the modelling and validate the findings. Samples were prepared using polyvinylalcohol, PVA (average Mw 85 k −124 k, 98–99% hydrolysed, Sigma-Aldrich, USA) as previously outlined.^34^ Briefly, crystalised PVA was mixed with deionised water, then heated and maintained at 95°C until a homogeneous solution formed, and then cooled at room temperature.

To evaluate the mechanical properties of the material, three samples of each PVA concentration, 5 and 10% by mass, were made for compression testing (Bose 3100, Framingham, MA). The PVA solution was poured into cylindrical moulds with diameter 16.7 ± 0.4 mm and underwent three freeze-thaw cycles. The height of each sample was then measured with a digital calliper, and the diameter assumed equal to the internal diameter of the mould. The samples were placed in contact with the load cell (22 n, Honeywell, Charlotte, NC), otherwise unconfined, and compressed by approximately 0.01 mm. Displacement control was performed using WinTest seven software (TA instruments, New Castle, DE). After 6 min, when the material had relaxed, a linear ramp was applied, compressing the sample by 0.2 mm at a rate of 1 mm s^−1^.

Force and displacement as a function of time was exported and scaled by surface area and initial height to obtain stress and strain, from which instantaneous Young’s modulus was calculated.

Samples for magnetomotive ultrasound imaging were also prepared using 5 and 10% by mass of PVA, and fine graphite powder (104206, Merck KGaA, Darmstadt, Germany). The graphite powder was added at 30 mg ml^−1^ of PVA solution—the particles increasing the ultrasound scattering. The solution was then poured into a resin mould (Gray V4, Formlabs Inc., Somerville, MA) measuring 10 × 10 × 25 mm. The mould had a small cylinder, radius 2.5 mm and length 10 mm, at the centre to create a hole through the cross-linked PVA cryogel. A cylindrical PVA insert of this length and diameter was produced, with the same composition except the addition of 2 kg magnetic nanoparticle solution (synomag-D micromod, Rostock, Germany) per m^3^ PVA solution. In total six phantoms were produced, three with 5% by mass PVA and three with 10%. All components underwent three freeze-thaw cycles to obtain physiological stiffness,^35^ and the cylinder was inserted into the hole after cross-linking.

### Magnetomotive ultrasound imaging

The phantoms were imaged using the MS-250 transducer, with a centre frequency of 21 MHz transducer with a preclinical ultrasound scanner (Vevo 2100, Visualsonics, Toronto, Canada). The transducer was positioned opposite a solenoid with the phantom between, with couplant gel providing acoustic contact with the probe. The solenoid, height 30 mm, 54 mm in diameter, including a pointed iron core with base diameter 27 mm, was operated at 2.5 Hz through a function generator and amplifier (Behringer EP4000, Willich, Germany), set to 16 or 20 dB. Data were collected for 2 s at 58 frames per second, the phantom position was then adjusted to acquire one replicate measurement of a different image plane.

### Magnetisation field

The first derivative in z of the magnetisation field (H) in the magnetic force expression, Equation 1, was calculated from experimental data. The magnetic field from the experimental setup; a solenoid (in-house construction) connected to an amplified (Behringer EP4000, Willich, Germany) harmonic signal (Agilent 3,3250A, CA) at 2.5 Hz was measured (HIRTS Magnetic Instruments Ltd. GM08, Cornwall, UK). A 20 cycle burst of 3 V peak–peak was amplified with the setting at 16 or 20 dB, matching the MMUS measurement. A hall probe (Transverse probe TP002) was positioned above the core tip and the analogue output was recorded to an oscilloscope. Root mean square of the recorded voltage was used to calculate the magnetisation field in Tesla. The probe was moved up to a height of 7 mm in steps of 1 mm, and three measurements were collected at each position. The data were normalised by the permeability of free space before fitting a cubic spline interpolant in the piecewise polynomial form.

## Finite element modelling

The mechanical response of a magnetic microbubble contacting an elastic solid was modelled using a finite element simulation software (COMSOL Multiphysics v. 5.6, Stockholm, Sweden).

### Geometry

**Figure 1.**
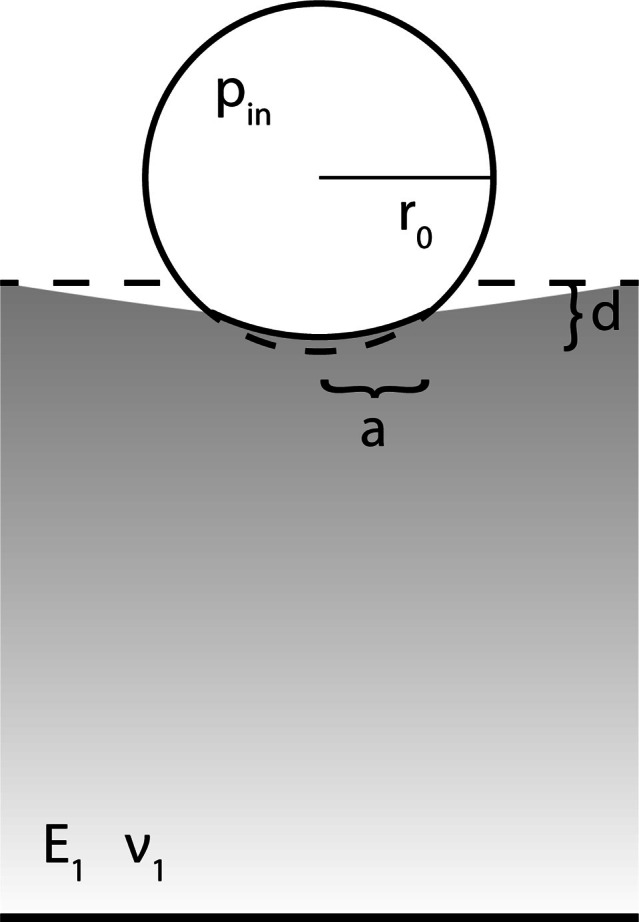
Cross-sectional view of model geometry after deformation of spherical shell with radius $r_{0}$ , internal pressure $p_{in}$ and a solid cylinder with Young’s modulus $E_{1}$ and Poisson’s ratio $\nu_{1}$ . Total deformation is d and the contact area covers an indent with a radius of a , that describes a circle perpendicular to the vertical symmetry axis. The bottom surface of the solid is fixed and its remaining boundaries are free. A spring foundation applied to the shell boundary prevented rigid motion of the sphere and was incrementally, non-linearly decreased while the force and pressure were ramped up. Regarding the bubble, only the shell was modelled explicitly using shell elements with a thickness of 2 nm. The internal pressure was applied on the boundary and was ramped up. Due to rotational symmetry, the geometry was created as a semicircle and a rectangle. Symmetry boundary conditions were applied on the axis of symmetry.

The magnetic microbubble was represented by a thin elastic shell that was subjected to a force. The bubble was forced into contact with an elastic barrier in order to study the contact pressure and deformations. The geometry is represented in Figure 1.

Due to rotational symmetry, the geometry was created as a semicircle and a rectangle rotated around the symmetry axis. Microbubbles were modelled as a thin shell surrounding a gaseous core. This was represented using shell elements, and a pressure boundary load applied to the shell.

### Contact and load definitions

Contact was defined using the penalty method, where the normal contact pressure, $T_{n}$ , is governed by the (overclosed) normal gap distance, $g_{n}$ , according to a conditional statement. The penalty factor was manually set to be suitable for bending dominated problems such as contact with shells,^36^ see Supplementary Material 1 for details. A contact pair was defined and the bottom half of the shell set as source and the top surface of the solid as destination. In the initial geometry, bubble and solid were separated by a gap and brought into contact by a force.

The magnetic force vector F **,** due to a gradient field, can be derived from Maxwell’s equations and is given by $F=\mu_{0}\left(M∙\nabla \right)H,$ under the assumption that curl of the field is negligible. Here, $\mu_{0}$ is the magnetic permeability of free space, M , the magnetisation, which is a non-linear function of the magnetisation field H , that asymptotically approaches a saturation value.^37^ This expression is valid assuming the electric field is zero, and that M≪H .

Given that the setup is an axisymmetric solenoid, and the region of interest is in the order of microns and centred about the symmetry axis, the magnetic force was assumed to be dominated by the axial component, and uniform throughout the volume of the shell. Furthermore, the magnetisation was estimated using the non-linear magnetisation data^38^ and the measured field at 1 mm separation from the solenoid core tip.

With these assumptions, the vector valued force simplifies to a scalar expression of the total force on one microbubble,

$$\begin{array}{c} F_{MB}=\mu_{0}M\left(H\right)m\frac{\partial H}{\partial z}. \end{array}(1)$$

Here M is the magnetisation by mass of iron for the magnetic nanoparticle suspension, which is a function of the magnetisation field H , m is the total mass of iron in a single bubble, and the first derivative in z of the magnetisation field H is taken at the point of interest. The magnetisation M for the corresponding field was estimated based on the magnetisation curve.^38^

The contact between a microbubble and an elastic solid resembles contact between an elastic sphere and half-space, which can be analytically solved using Hertz contact theory.^33^ It relates the force and deformation to properties of the sphere and half-space. Normal contact pressure p(r) is distributed on a circle of radius a according to

$$\begin{array}{c} p\left(r\right)=p_{0}{\left(1-\frac{r_{0}^{2}}{a^{2}}\right)}^{\frac{1}{2}},\;(2) \end{array}$$

where $p_{0}=3F/2\pi a^{2}$ , F is the applied load, $r_{0}$ is sphere radius. The contact pressure distribution is also influenced by Young’s moduli E and Poisson’s ratios ν of the contacting bodies, see Supplementary Material 1. This theory was used to formulate expressions for contact area, contact pressure and displacement, to compare with outputs from the finite element modelling.

### Structural mechanics

The bubble shell was modelled as isotropic and linearly elastic with a Young’s modulus, Poisson’s ratio, density and thickness of 100 MPa, 0.499, and 1100 kg m^-3^ and 2 nm respectively.^39^ The mechanical properties of the bubble also depend on the encapsulated gas, or rather on the pressure it exerts. The equilibrium pressure inside the bubble $p_{in}$ depends on the size and surface tension, σ , of the bubble, according to the Young-Laplace equation

$$\begin{array}{c} p_{in}=2\frac{\sigma }{r_{0}}.(3) \end{array}$$

The inverse relation with radius, $r_{0}$ , leads to substantial overpressure in microbubbles where the radius is typically less than 10 microns.

The solid was also modelled as isotropic and linearly elastic, with Young’s modulus, Poisson’s ratio and density of 24 kPa, 0.42,^35^ and 1300 kg m^-3^ respectively.^40^

### Solver configuration and convergence analysis

An auxiliary sweep was used to stabilise the stationary analysis. The swept parameter controlled a spring foundation, force and pressure, and was ramped up incrementally from zero to one.

An ordered hexahedral mesh was used for the solid, which was refined in the region with potential contact. The mesh was incrementally refined until convergence was achieved, based on the centre point displacement. The solution was considered converged when further refinement did not change the third significant digit.

## Results

Here, we report the results from the experimental imaging and FEA.

### Contrast-enhanced imaging to detect lymph nodes

**Figure 2.**
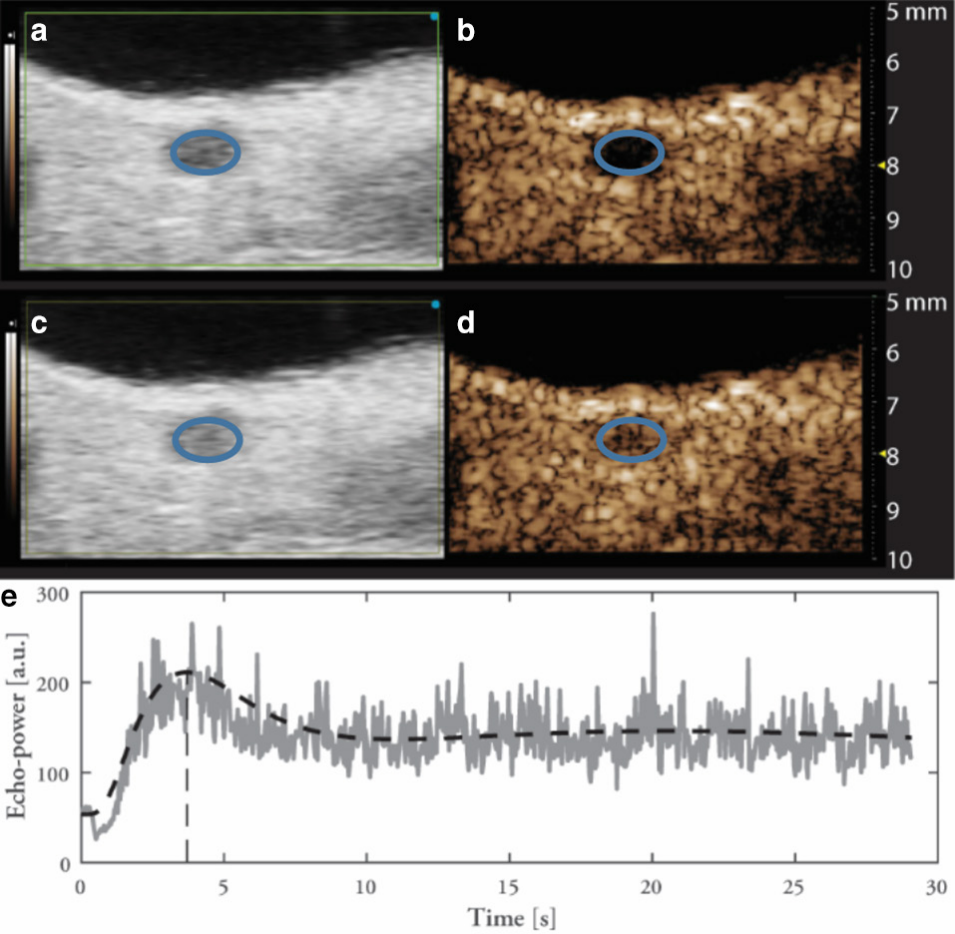
Ultrasound B-mode (panels A and C) and contrast mode images (**B and D**) of lymph node pre-contrast administration and at peak enhancement on the top and second row respectively. The lymph node, indicated by an oval in panels A through D, is distinguishable from the background in the B-mode images (left), as a hypoechoic region. The same region is clearly void of non-linear signal in the absence of contrast agent, see panel B, but shows a strong signal post-injection, panel D. The filling of the region of interest outlining the lymph node is shown in panel E, showing peak enhancement 3.7 s post-injection.

Contrast-enhanced ultrasound imaging of the inguinal lymph node was performed using magnetic microbubbles, see Figure 2.

### Influence of Young’s modulus and excitation force on magnetomotive imaging

**Figure 3.**
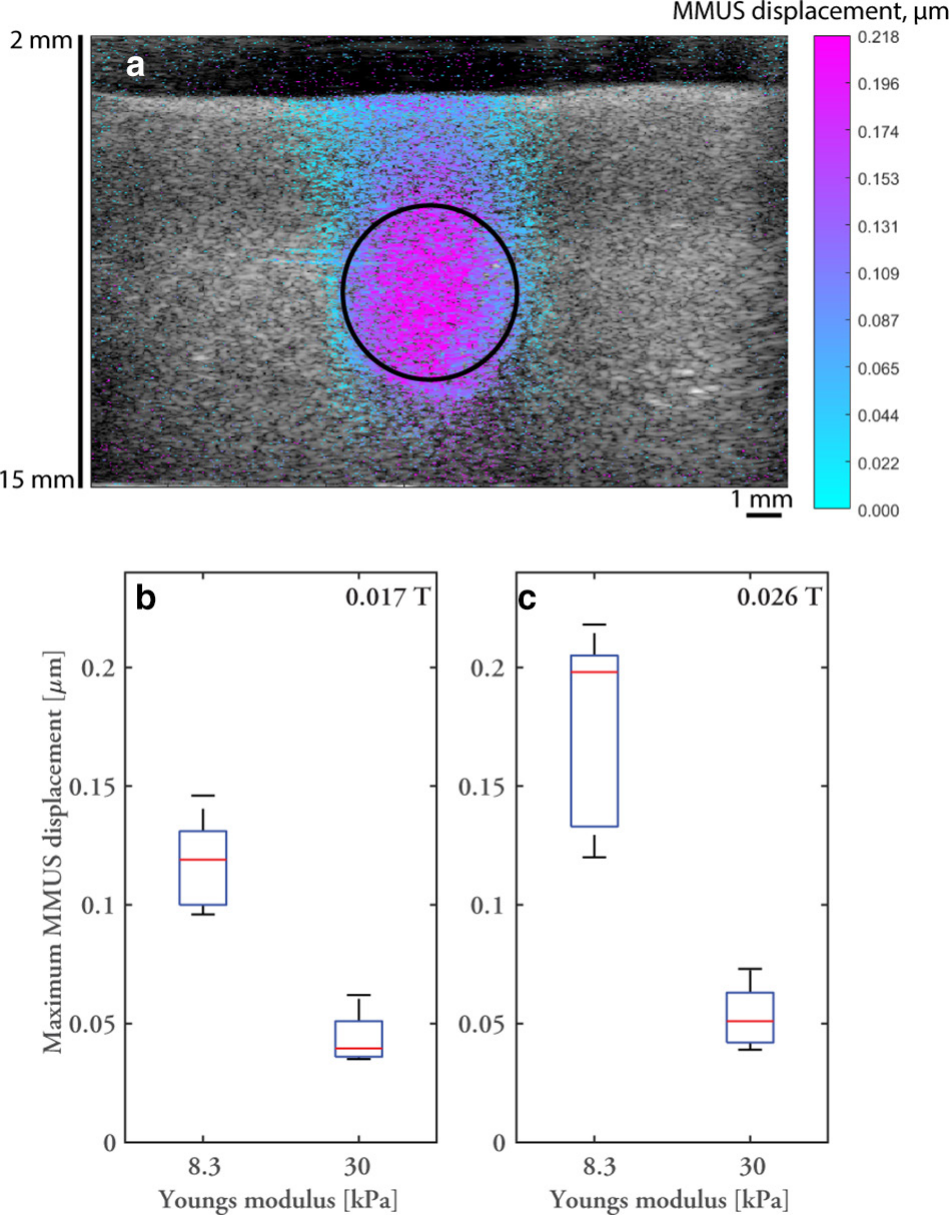
MMUS imaging: MMUS displacements were detected predominately in the insert containing magnetic nanoparticles, see image in panel A of 5% PVAc phantom at high excitation current, 3 V peak-peak amplified by 20 dB. The size and approximate position of the insert is indicated by a circle. B shows the maximum displacement in six phantoms with PVA concentrations, 5 and 10%, for two excitation settings, such that the magnetic flux density at the centre of the insert was approximately 0.017 T and 0.026 T respectively. Magnetic flux density, or more precisely magnetisation field, is an important factor in determining the magnetic force, see Equation 1, and the different concentrations of PVA produce cryogels with distinctly different stiffnesses, or Young’s modulus of 8.3 and 30 kPa respectively. Each box represents six measurements taken on three phantoms in two image planes. The centre line represents the median, box edges are 25th and 75th percentile and whiskers show the full range of values recorded. MMUS, magnetomotive ultrasound; PVA, polyvinyl alcohol.

In addition to contrast-enhanced imaging of magnetic microbubbles, MMUS imaging using magnetic nanoparticles was performed, see Figure 3.

Young’s modulus of the phantom material distinctly influences maximum MMUS displacement as does excitation field, which is a factor determining the magnetic force.

**Figure 4.**
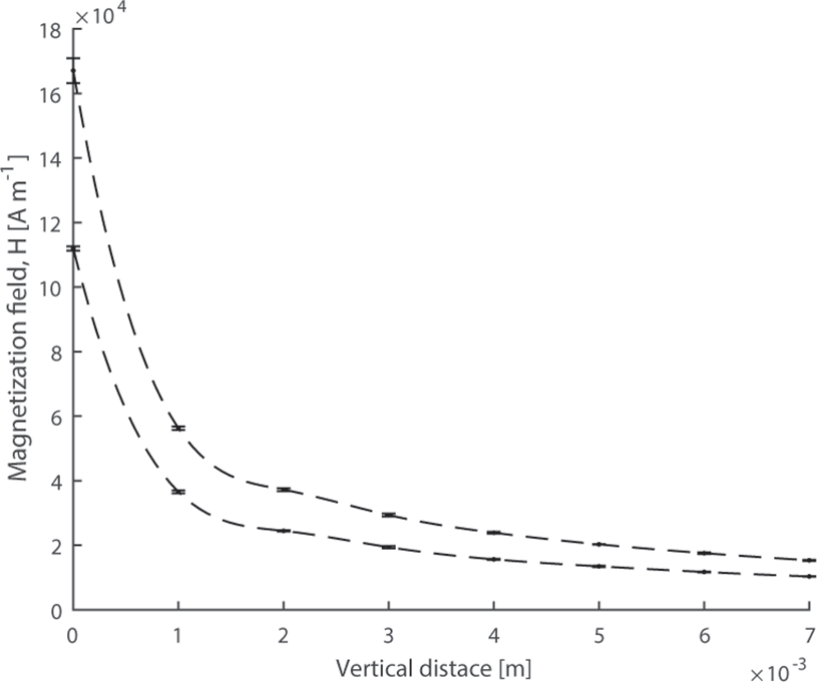
Field measurements along the symmetry axis of the solenoid for two amplification settings, 20 dB (top) and 16 dB (below). Each point represents the mean of three measurements, and error bars indicate standard deviation. The first derivative was obtained from a cubic spline interpolant in the piecewise polynomial form, shown as a dashed line.

Field measurements on the solenoid, see Figure 4, were used to calculate the magnetic force.

The total force was calculated from the top curve in Figure 4 at 1 mm separation from the tip and 20 dB amplification setting according to Equation 1 as 1.0 pN, given a magnetisation of 74.5 Am^2^kg^−1^ Fe based on the value for the magnetic particles at the corresponding magnetisation field, and 0.71 pg Fe per microbubble.^19^ This force magnitude was used as an input to model the mechanical response of a magnetic microbubble contacting an elastic solid.

### Finite element modelling of magnetic microbubble

Convergence was reached with a mesh consisting of 1220 boundary elements and 576 domain elements with average quality 0.87 (and minimum 0.5) as evaluated by skewness. This mesh configuration was used throughout.

**Figure 5.**
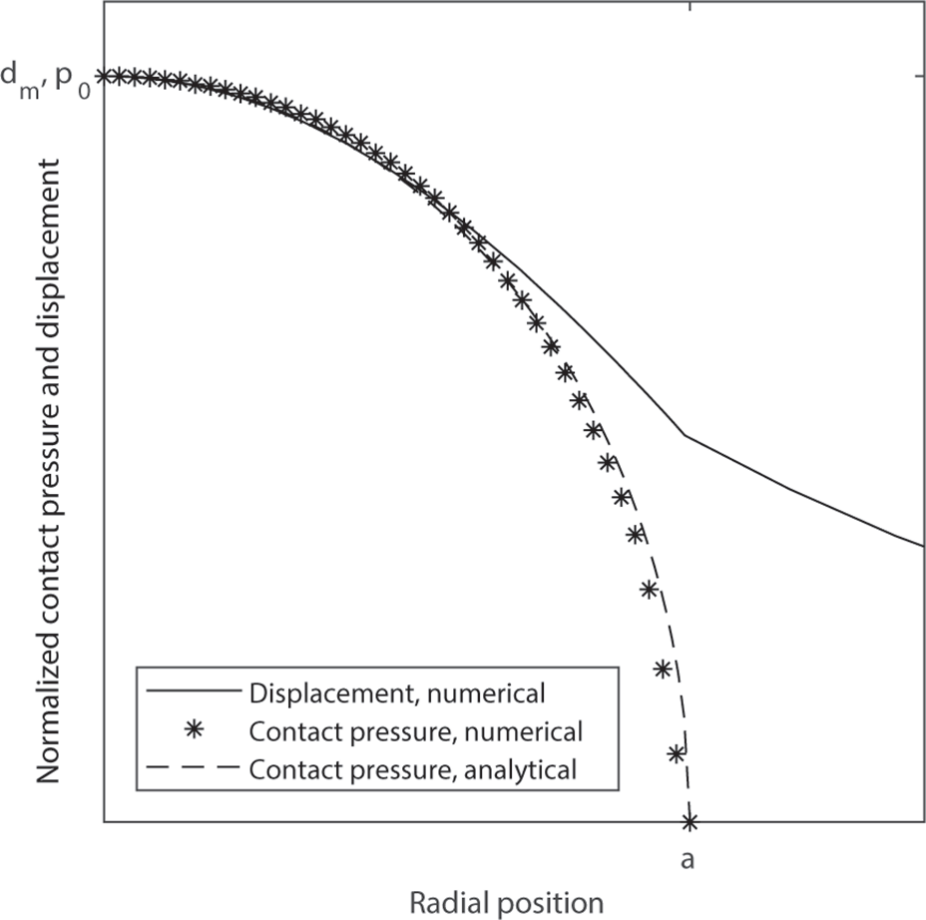
Finite element modelling of tissue deformation due to magnetic microbubble motion: normalised contact pressure (asterisk) and solid displacement (solid line) occurring due to a magnetic microbubble moving under a magnetic field. The analytical contact pressure according to Hertz contact theory is shown as a dashed line. Each variable was normalised to its maximum value.

The finite element modelling outputs illustrate the contact pressure and deformation of the elastic solid due to a magnetic microbubble being forced into it, see Figure 5.

The contact pressure is highest towards the centre of the bubble and drops to zero at the radius where the bubble surface is no longer in contact with the solid. Displacement of the surface of the solid is also largest at the centre point and decreases with increasing radial distance from the centre. Note that the deformation of the solid extends outside of the contact area.

**Figure 6.**
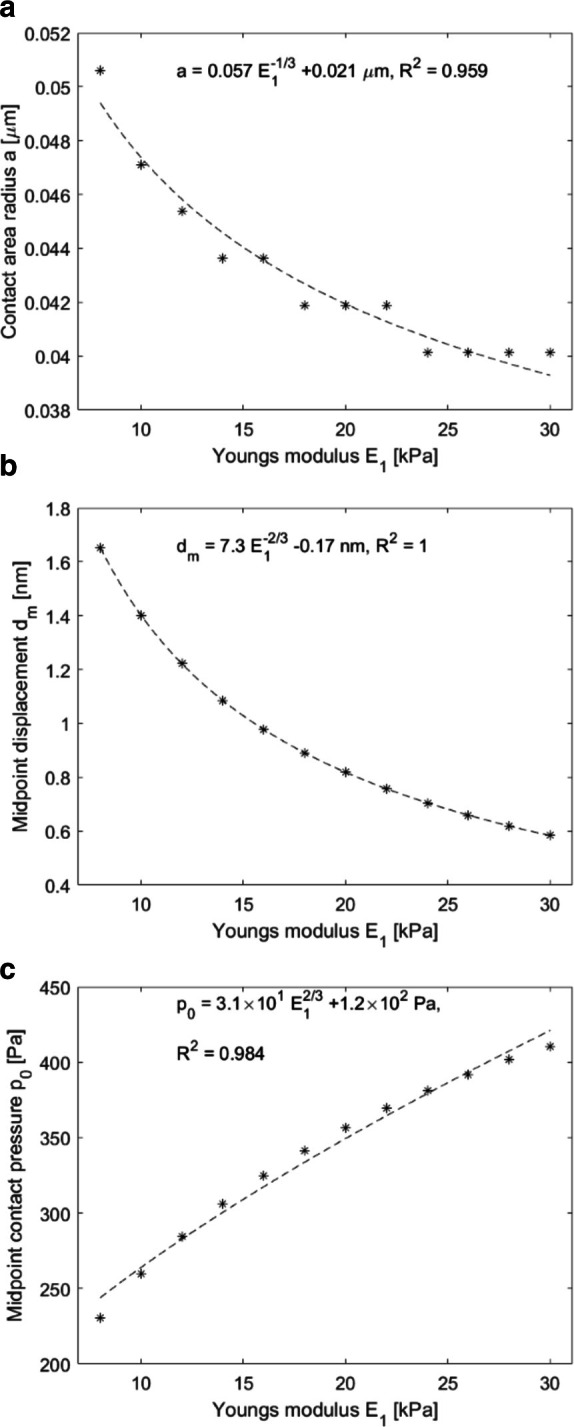
Contact area radius (**A**), midpoint displacement (**B**) and midpoint contact pressure (**C**) outputs for a magnetic microbubble contacting an elastic solid with Young’s moduli ranging from 8 to 30 kPa. Bubble radius was 1.05 µm, and force was 1.0 pN. Curves fitted based on the Hertz contact theory.

The trends in contact area, displacement and contact pressure were explored in response to changes in the elasticity of the solid, bubble radius and force magnitude. First, the response variables were examined for Young’s modulus of the solid ranging from 8 to 30 kPa, see Figure 6.

The contact area and displacement decreases with increasing Young’s modulus, while contact pressure at the midpoint increases. The trend of decreasing displacement with increasing Young’s modulus was also observed in the MMUS measurements, see Figure 3b. Notably, the increase in Young’s modulus leads to a decrease in displacement, which is counterbalanced by an increase in contact pressure. The data were found to closely follow Hertz contact theory and as such, this theory was used to perform curve fits to the data points.

**Figure 7.**
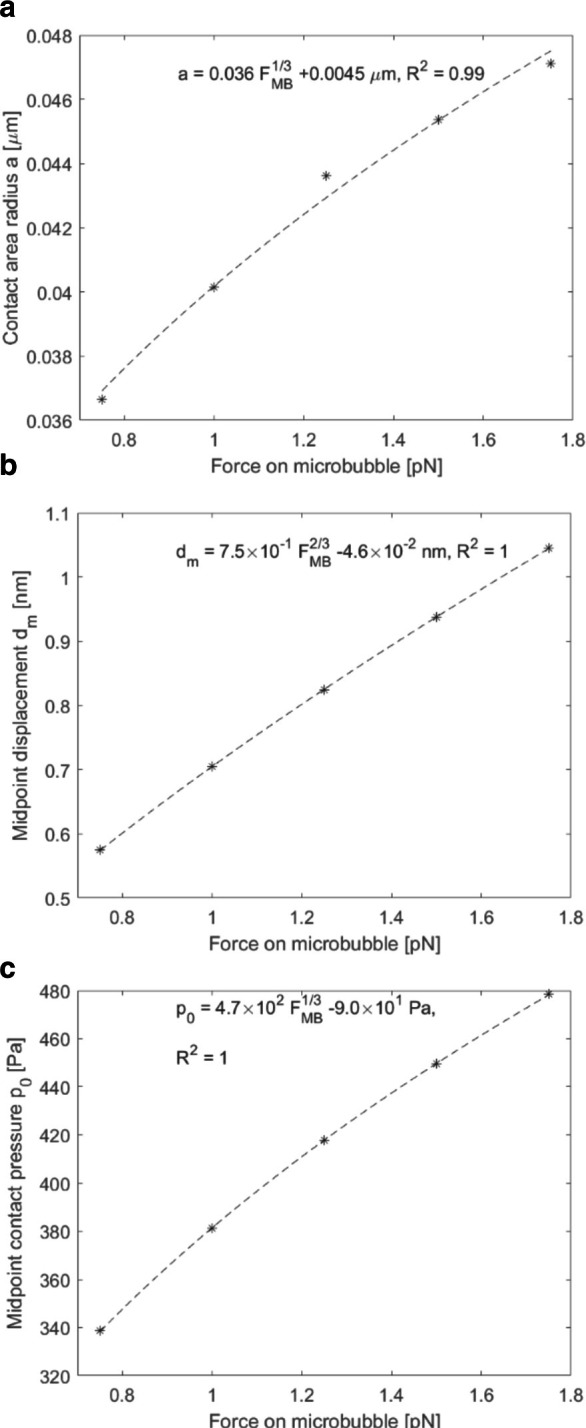
Contact area radius (**A**), midpoint displacement (**B**) and midpoint contact pressure (**C**) outputs for bubble radii ranging from 1 to 1.5 µm. Young’s modulus of the solid was 24 kPa, and force magnitude was 1.0 pN. Fitted curves based on the Hertz contact theory.

Similarly, the response to changing the bubble radius was modelled, see Figure 7.

When the bubble radius increases, the contact area also increases, while displacement and contact pressure at the midpoint decrease. Thus, a smaller bubble induces a larger displacement in the tissue, but the area of force transferral is reduced. In comparison, MMUS requires a sufficiently large displacement amplitude, as well as region, to allow robust detection using high resolution ultrasound imaging.

**Figure 8.**
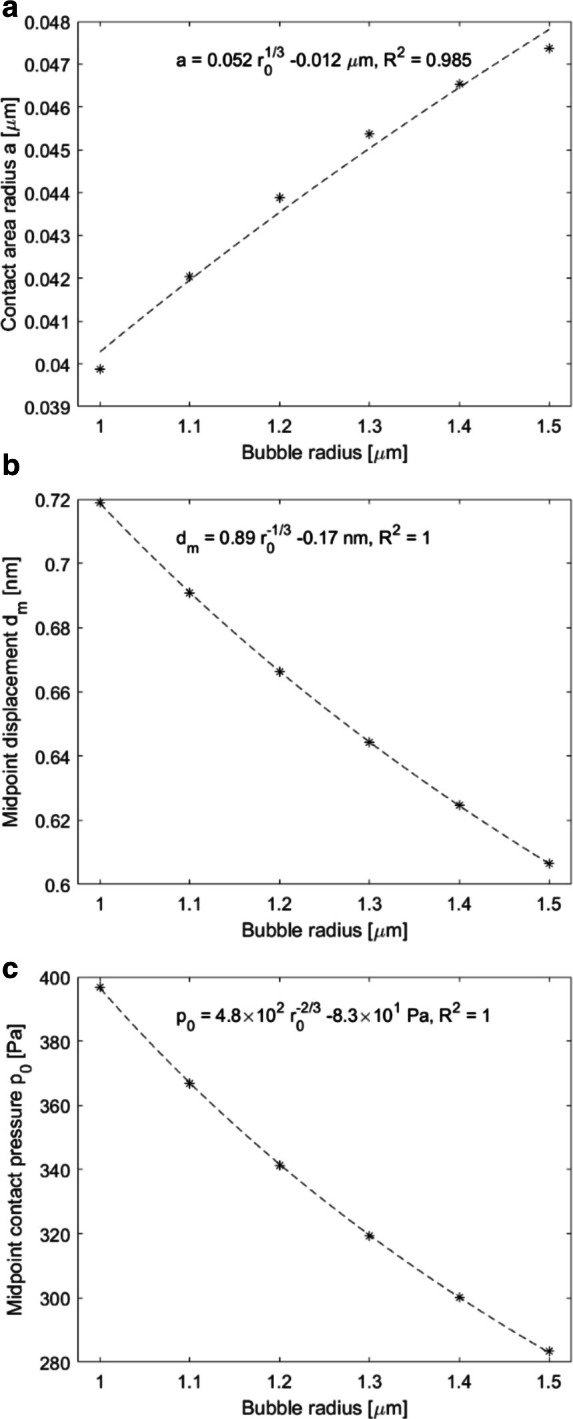
Contact area radius (**A**), midpoint displacement (**B**) and contact pressure (**C**) outputs for total force magnitude ranging from 0.75 to 1.75 pN. Bubble radius was 1.05 µm, and Young’s modulus of the solid was 24 kPa. Fitted curves are based on the Hertz contact theory.

The influence on each parameter of interest in response to total force are presented in Figure 8.

Increasing the force magnitude simultaneously increases contact area, midpoint displacement and midpoint contact pressure. These results are in agreement with the trend also observed in the MMUS data, Figure 3, namely that increasing the applied magnetic force increases the displacement magnitude.

## Discussion

Medical imaging is integral to cancer,^10^ diagnosis, staging, and treatment planning.^8^ Information about the location and status of lymph nodes is essential to guide treatment planning and holds the potential to allow the surgeon to excise tissue in a more conservative manner, reducing the invasiveness or extent of excision. This has obvious associated benefits to the patient’s quality of life both in short- and long-term. However, for CRC, targeted resection is not always possible due to the absence of robust methods for obtaining reliable and accurate lymph node assessment.^41^ Contrast- enhanced ultrasound using microbubbles^19^ and MMUS imaging^26^ have been separately demonstrated as feasible methods for lymph node detection. It has been hypothesised that combining the methods could have synergistic effects and enhance sensitivity.^42^ Here, we explore the feasibility of combining both techniques by using magnetic microbubbles for CE-MMUS. Below, we discuss the potential of this new technique for enhanced lymph node imaging.

Contrast-enhanced imaging of the inguinal lymph node *in vivo* was demonstrated using magnetic microbubbles, see Figure 2. This demonstrates that accumulation of the magnetic contrast was achieved, which is required to enable lymph node CE-MMUS imaging. This was achieved even without magnetic targeting, which could contribute to increased retention if employed.^24^ Furthermore, the non-linear response was detectable for the magnetically functionalised microbubble suspension, indicating that this property was preserved. Although functionalisation may affect the mechanical properties of microbubbles, and thereby alter their non-linear acoustic response,^43^ this does not appear to be the case for this method of magnetic functionalisation.^24^ Importantly, this means that the acoustic properties of microbubbles are preserved, and can be exploited in CE-MMUS using magnetic microbubbles.

The experimental section also demonstrated MMUS and the inherent link to tissue properties. MMUS displacement magnitude is governed by many factors, including the elastic properties,^29^ and the magnitude of the magnetic excitation force,^44,45^ which is shown in Figure 3. Even small magnetomotion, less than a micrometre, can be distinguished from noise due to its characteristic frequency and phase,^46^ and linking the MMUS displacement amplitude to elastic properties of the tissue^30^ is a strong motivation for pursuing the implementation of CE-MMUS. The use of magnetic microbubbles could add new functionality to MMUS by allowing lymphatic drainage kinetics to be visualised, or due to the nature of the microbubbles themselves, present new methods of interrogating tissue where the response could be detected via ultrasound imaging. In MMUS, motion is generated from within a viscoelastic medium, while microbubbles are present within a liquid medium and influence a solid medium through direct contact. In this regard, using magnetic microbubbles in CE-MMUS rely on a different mechanism of force transferral between the contrast agent and the tissue. Validating the finite element findings experimentally is a crucial challenge moving forward.

FEA was used to study the magnetomechanical interaction between a magnetic microbubble with a tissue interface. The force estimated by the model at 1 mm separation is 1.0 pN, which agrees with existing literature.^47^ The main results demonstrate the ability of the magnetic force to attract a microbubble towards the interface, and to deform it through contact (Figure 5). This has two implications for CE-MMUS.

First, the ability to attract bubbles through a magnetic gradient force demonstrates how a static magnetic field can be used to manipulate loaded bubbles. This is instrumental to magnetic targeting, whereby loaded microbubbles are retained in a region of interest. This property could be used in order to accumulate microbubbles for MMUS or elastography. Retention of magnetic microbubbles though a gradient field has been shown experimentally, with higher iron loading correlating to a higher degree of retention.^24^

Second, induced deformation and displacement is the basis of the contrast mechanism of MMUS. These results therefore illustrate how magnetic microbubbles could act as a contrast agent in MMUS. Furthermore, the similarity with the analytical contact model^33^ lets us examine the trends across a range of values for important variables such as Young’s modulus of the solid, radius of the bubble, as well as magnetic force magnitude. They point to an inverse relation between displacement and tissue elasticity (Figure 6). Decreasing displacements with increasing Young’s modulus can also be seen in the experimental data, Figure 3. As previously demonstrated,^28^ MMUS is capable of indicating the presence of a nanoparticle-based contrast agent in tissue. Using a frequency- and phase-sensitive algorithm^34^ and an automatic phase interval selection criterion,^26^ the region containing contrast agent is clearly distinguishable from the background with larger magnetomotion, and clearer delineation in the softer material. While the experimental work was limited to two concentrations of PVA, producing two different Young’s moduli, FEA was used to demonstrate trends across a range of parameter values. The inverse dependency of magnetomotive displacement on Young’s modulus, also reported by Levy and Oldenburg,^48^ again implies a possibility to infer mechanical properties.^28–30^

In addition to Young’s modulus, changing the bubble radius was also found to affect the deformation, with larger displacements indicated for smaller radii, Figure 7. However, reducing the radius also decreased the contact area of an individual bubble, such that this displacement occurred over a smaller region. In other words, decreasing the bubble size resulted in increased amplitude of motion in a smaller region. To robustly detect motion in MMUS, both the amplitude of displacement and the size of the region that is being displaced have to be sufficiently large. The implications for CE-MMUS are that bubble size will play a crucial role in performance and optimisation and should therefore be carefully considered.

The total force acting on a magnetic microbubble also influenced the response in terms of expected tissue displacement, Figure 8. Increasing the force intuitively increased all response variables. In practice, the magnetic force can be increased by increasing the strength of, or decreasing the distance to the magnet, or by improving the magnetic properties or increasing the concentration of the contrast agent. For CRC, the first two properties, strength and distance, are constrained by the endoscopic probe that would be used to apply the magnetic field. Thus, in practice, the force magnitude would be governed by the contrast agent. Interestingly, we can calculate an equivalent iron concentration of a collection of magnetic microbubbles and compare this to the concentration used in the phantom imaging. Assuming an iron loading of 0.71 pg Fe per microbubble, and a (relatively low^49^ concentration of 1.1 × 10^12^ MB m^−3^ (or, equivalently, 1.1 × 10^6^ MB ml^−1^) of the magnetic microbubbles,^24^ the equivalent iron concentration is 39% of the 2 kg Fe m^−3^ that was used in the phantom imaging. This is also comparable to the iron concentration of 0.92 kg Fe m^−3^ that has been imaged using MMUS in lymph tissue,^50^ indicating that bubble loading could achieve sufficient iron concentration for imaging.

There are different numerical methods for contact evaluation including penalty and Augmented Lagrangian. The penalty method offers comparatively fast computations and smooth convergence, and was used in the analysis. The drawback is reduced accuracy in contact distribution compared to the Augmented Lagrangian method. The numerical and analytical results presented here are both based on frictionless contact mechanics. This is a reasonable simplification for this model considering that the magnetic force acts in the normal direction, making tangential stresses practically inconsequential. However, extending the model to account for a magnetic force at an angle to the normal and viscous drag in a fluid would be more realistic for a clinical setting, and in that case accounting for friction would be relevant. The emerging trends from the FEA are of interest but note that the absolute values are difficult to validate experimentally. The force estimated by the model is in agreement with existing literature^47^ and the model outputs were also validated against an analytical model.

For a single microbubble, the situation resembles an elastic sphere in contact with an elastic solid, a case that is analytically described by Hertz contact theory.^33^ The main difference is that a microbubble is not homogeneous, but consists of a thin shell and a gaseous core. For small deformations and quasi-static conditions, this does not cause a significant deviation from Hertz theory, and the bubble can be treated as an elastic sphere with unknown bulk properties. This results in excellent agreement between FEA and the analytical model, with R^2^ ranging from 0.959 to 1.000.

For large deformations, the pressure inside the bubble would be expected to vary significantly. Any changes to the bubble volume can contribute to changes in the internal pressure, a relation that can be described by the polytropic process equation.^31,32^. For small volume changes, such as those occurring in response to the magnetic force, this contribution can be considered negligible.

During insonation, the pressure and volume changes significantly and dynamically. Interactions between microbubbles and vessel walls have been studied in the context of sonoporation.^25,31^ These oscillations occur on a much faster time scale than the magnetic force, which poses challenges in regards to simulating both processes together. In this model, we have isolated the interactions due to the magnetic force. Future work should consider the influence of both acoustic and magnetisation field on the magnetic microbubble.

The dynamics of an assembly of microbubbles is affected by interactions between individual bubbles. Since the magnetic field can act to concentrate the bubbles in a region, and the probability and strength of interaction increases with decreasing distance, this is another important aspect to consider.

Further research is needed to understand the dynamic interactions of microbubbles and specifically during simultaneous magnetic excitation and insonation. Experimental validation is of particular interest, but additional FEA would also be beneficial to predict and model these dynamics. Challenges associated with the different time scales, with frequencies in the range of Hz and MHz respectively can be addressed in FEA. For example, by separating the problem into a quasistatic and a dynamic component.

The robust detection of lymph nodes and characterisation of tissue mechanical properties are key to accurate cancer staging. Theoretically, combining contrast enhanced- and MMUS imaging has the potential to achieve this. However, to realise it requires the magnetic microbubbles to accumulate in the region of interest where they can be magnetically manipulated to interact with the tissue. We have demonstrated how a magnetic gradient field can act to attract magnetic microbubbles and cause deformations through contact with an interface, and how the degree of deformation depends on properties such as bubble radius and tissue elasticity. Inversely, tissue mechanical properties can be inferred from the characteristics of mechanical vibrations induced by a magnetic excitation,^30^ and such information can be used to help determine tissue status.^13^ Theoretically, these results illustrate that these foundations of CE-MMUS in lymph node detection and localisation are realisable. In practice, there are more interactions to consider with regards to magnetic microbubbles, such as the possibility of retention, sonoporation and targeted delivery. Together with the results presented here, these capabilities indicate prognostic and therapeutic potential for CE-MMUS.

## Supplementary Material

bjr.20211128.suppl-01
