# Supplementary material for: Modelling of magnetic microbubbles to evaluate contrast enhanced magnetomotive ultrasound in lymph nodes – a pre-clinical study
Source: Br J Radiol. 2022 May 19;95(1135):20211128. doi: 10.1259/bjr.20211128 (PMC10996324; doi:10.1259/bjr.20211128)
Supplement: bjr.20211128.suppl-01 [file bjr.20211128.suppl-01.docx]

Appendix

1. Penalty method

In the penalty method of contact, the normal contact pressure, $T_{n}$, is governed by the (overclosed) normal gap distance, $g_{n}$, according to a conditional statement

$$\begin{aligned} T_{n}=\left\{ \begin{matrix} -p_{n}g_{n}, & g_{n}\leq0 \\ 0, & g_{n}>0 \end{matrix} \right.. \#A1 \end{aligned}$$

Here $p_{n}$ is the penalty factor. The penalty is determined by Young’s modulus, $E$, and minimum element size, $h_{min}$, and a penalty factor multiplier, $f_{p}$ according to

$$\begin{aligned} p_{n}=f_{p}\frac{E}{h_{min}}, \#A2 \end{aligned}$$

with $f_{p}$= 2x10^-5^.

1. Hertz contact theory

Hertz contact theory relates the contacting radius to the applied force,$F$, sphere radius, $r_{0}$, and Young’s moduli and Poisson’s ratios, $E$ and $\nu$ according to

$$\begin{aligned} a^{3}=\frac{3r_{0}}{4}\left( \frac{1-\nu_{1}^{2}}{E_{1}}+\frac{1-\nu_{2}^{2}}{E_{2}} \right). \#A3 \end{aligned}$$

The maximum contact pressure, $p_{0}$ is given by

$$\begin{aligned} p_{0}=\frac{3F}{2\pi a^{2}}, \#A4 \end{aligned}$$

and the sphere and contact radii are finally related to the displacement $d$

$$\begin{aligned} a=\sqrt{r_{0}d}. \#A5 \end{aligned}$$

Table A1: Summary of parameters of finite element model

| Parameter | Value | Reference/motivation |
| --- | --- | --- |
| Magnetisation, M | 74.5 A m^2^ kg^-1^ Fe | Based particle suspension^1^ and measured magnetization field at 1 mm separation. |
| Force on microbubble, F_MB_ | 1.0 pN | Calculated using data in Figure 4 for 20 dB at 1 mm separation and magnetisation M. |
| Young’s modulus of elastic solid, E_1_ | 24 kPa | From measurement on PVA |
| Poissons ratio of elastic solid, ν_1_ | 0.42 | ^2^ |
| Density of elastic solid | 1300 kg/m^3^ | ^3^ |
| Young’s modulus of shell | 100 MPa | ^4^ |
| Poisson’s ratio of shell | 0.499 | ^4^ |
| Density of shell | 1100 kg/m^3^ | ^4^ |
| Thickness of shell | 2 nm | ^4^ |
| Surface tension of shell, σ | 25 mN/m | ^5^ |
| Initial bubble radius, r_0_ | 1.05 μm | ^6^ |
| Pressure, p_in_ | -(2σ/r_0_) | Young-Laplace equation |

References

1. Yuan Y, Borca-Tasciuc DA, Rende D, Ozisik R, Altan CL, Bucak S. Effect of surface modification on magnetization of iron oxide nanoparticle colloids. Langmuir. 2012;28(36):13051-59.

2. Fromageau J, Brusseau E, Vray D, Gimenez G, Delachartre P. Characterization of PVA cryogel for intravascular ultrasound elasticity imaging. IEEE Transactions on Ultrasonics, Ferroelectrics, and Frequency Control. 2003;50(10):1318-24.

3. Stasko J, Kalniņš M, Dzene A, Tupureina V. Poly (vinyl alcohol) hydrogels. Proceedings of the Estonian Academy of Sciences. 2009;58(1).

4. Leaute G, McLaughlan J, Harput S, Cowell D, Freear S. Comsol modelling of non-spherical microbubble dynamics near a soft membrane. 2012 IEEE International Ultrasonics Symposium. 2012:2286-89.

5. Borden MA, Longo ML. Dissolution Behavior of Lipid Monolayer-Coated, Air-Filled Microbubbles:  Effect of Lipid Hydrophobic Chain Length. Langmuir. 2002;18(24):9225-33.

6. Beguin E, Bau L, Shrivastava S, Stride E. Comparing strategies for magnetic functionalization of microbubbles. ACS Applied Materials & Interfaces. 2019;11(2):1829-40.
